# Symbiotic Fungus Affected the Asian Citrus Psyllid (ACP) Resistance to Imidacloprid and Thiamethoxam

**DOI:** 10.3389/fmicb.2020.522164

**Published:** 2020-12-16

**Authors:** Tuyong Yi, Ling Lei, Ling He, Jianglan Yi, Lingguo Li, Liangying Dai, Yanyun Hong

**Affiliations:** ^1^Hunan Provincial Key Laboratory for Biology and Control of Plant Pests, College of Plant Protection, Hunan Agricultural University, Changsha, China; ^2^College of Life Science and Technology, Beijing University of Chemical Technology, Beijing, China

**Keywords:** Asian citrus psyllid (ACP), symbiotic fungus, resistance, imidacloprid, thiamethoxam

## Abstract

The Asian citrus psyllid (ACP), *Diaphorina citri* (Kuwayama) (Hemiptera: Liviidae), is a notorious Rutaceae plant pest. Frequent and extensive use of pesticides has resulted in severe insecticide resistance in ACP populations. Fully understanding the mechanism of ACP resistance to pesticides is vital for us to control or delay the development of resistance. Therefore, we compared the difference in resistance to imidacloprid and thiamethoxam between Hunan (Yongzhou, Chenzhou) and Guangdong (Guangzhou) ACP populations and analyzed the correlations between the resistance level and genes and symbiotic fungi. The results showed that the resistance of the Guangdong ACP population to imidacloprid and thiamethoxam was lower than that of Hunan ACP population, and the relative expression of genes associated with P450 mono-oxygenase and acetylcholinesterase was significantly lower in the Guangdong ACP population than in Hunan ACP population. The differences of mean relative abundances of four symbiotic bacteria among three populations were marginally significant; however, the mean relative abundance of 16 fungi among three populations was significantly different, and positive linear correlations were observed between the resistance level and two fungi (*Aspergillus niger* and *Aureobasidium pullulans*) and two genes (*CYP4C70* and *CYP4DB1*). Negative correlations were only observed between the resistance level and two fungi (*Golubevia pallescens* and *Acremonium sclerotigenum*). Moreover, four fungi were unique to the Chenzhou population which was the highest resistance to imidacloprid and thiamethoxam. These findings suggested the P450 mono-oxygenase and symbiotic fungi together affected ACP resistance to imidacloprid and thiamethoxam. In the future, we may use environmental *G. pallescens* and *A. sclerotigenum* to control or delay ACP resistance.

## Introduction

Chemical control is the most important and widely used measure in controlling most agricultural pests (Dang et al., 2017). The resistance level of pests is increasing with the intensive and frequent use of insecticides, reducing the efficiency of pesticides, increasing costs in the agricultural industry, and causing serious environmental pollution and potential risks to human health. Fully understanding the mechanisms of pesticide resistance is vital for us to control or delay the development of resistance. Therefore, the analysis of metabolic enzyme activity, application of insecticide synergists, and expression of genes have been used to explore the mechanisms of pesticide resistance. Wu et al. (2018) found that the *CYP6ER1* gene in *Drosophila melanogaster* conferred resistance to imidacloprid, thiamethoxam, and buprofezin. Garrood et al. (2016) found that the *CYP6ER1* gene provided the brown planthopper (BPH) (*Nilaparvata lugens*) resistance to imidacloprid and ethiprole. Increased activity and reduced sensitivity of acetylcholinesterase affected oriental migratory locust resistance to the organophosphate insecticide malathion (Yang et al., 2009). Liu et al. (2005) confirmed that nicotinic acetylcholine receptor (*nAChR*) genes, as targets of neonicotinoid insecticides, affected BPH resistance to imidacloprid. Overall, most studies on insecticide resistance mechanisms have focused on evolutionary changes in pest insect genomes, such as the alteration of pesticide target sites, increase in pesticide excretion rates, and upregulation of ATP binding protein and metabolic enzymes, including general esterase, glutathione *S*-transferase, and cytochrome P450 mono-oxygenase (Cao et al., 2008; Kikuchi et al., 2012; Wei et al., 2015; Safi et al., 2017; Tian et al., 2018; Wang et al., 2019).

In fact, insects are multiorganismal symbionts and harbor many microbes that also mediate insecticide resistance (Douglas, 2015). An early study found that *Pseudomonas melophthora*, an obligate symbiont, helped an insect host (apple maggot, *Rhagoletis pomonella*) degrade 6 insecticides (Boush and Matsumura, 1967). The densities of symbiotic bacteria *Arsenophonus*, *Rickettsia*, and *Wolbachia* from the Q biotype whitefly associated with the host’s susceptibility to thiamethoxam, imidacloprid, pyriproxyfen, and spiromesifen (Ghanim and Kontsedalov, 2009). The symbiotic bacteria *Burkholderia* mediated the resistance of its insect host, *Riptortus pedestris*, to the organophosphate pesticide fenitrothion by degrading the pesticide (Kikuchi et al., 2012). Cheng et al. (2017) found that the gut symbiont *Citrobacter* sp. helps the host insect fruit fly *Bactrocera dorsalis* degrade the organophosphate insecticide trichlorphon. Pang et al. (2018) found that *Arsenophonus* strains (S-type *Arsenophonus*) in the BPH negatively affected the insecticide resistance of the host by downregulating xenobiotic metabolism and increasing amino acid accumulation.

In addition to the many studies focused on bacteria, the degradation of pesticides by fungi has also been explored. Marecik et al. (2008) found that *Volutella ciliata* degraded atrazine in soil. *Cladosporium cladosporioides* displayed maximum degradation of chlorpyrifos in soil (Bisht et al., 2019). The symbiotic fungus (*Candida lipolytica*) of the BPH (*N. lugens*) participated in resistance to imidacloprid (Li et al., 2010). Symbiotic yeast (*Symbiotaphrina kochii*) helps host cigarette beetles (*Lasioderma serricorne*) resist toxins (Dowd and Shen, 2011). *Aspergillus fumigatus* plays an important role in azole resistance (Howard et al., 2006). *Aspergillus flavus* can produce terreulactone with antiacetylcholinesterase activity and phosphohydrolases to help pest host detoxification to pesticides (Rudramurthy et al., 2019). Moreover, a previous study showed that *Thysanophora penicillioides* and *Dothideomycetes* affected the sensitivity of the Q biotype whitefly to *Beauveria bassiana* (a biological pesticide) (Hong et al., 2016). Therefore, microbes can influence host insect resistance to pesticides.

The Asian citrus psyllid (ACP), *Diaphorina citri* (Kuwayama) (Hemiptera: Liviidae), is a notorious Rutaceae plant pest. The ACP, a phloem-sap feeding pest, directly causes nutrient depletion within the plant, affecting the growth and death of young foliage at high population densities (Gottwald, 2010). Furthermore, ACP secretes honeydew, reducing photosynthesis and resulting in sooty mold (Grafton-Cardwell et al., 2013). Moreover, ACP is a natural vector of *Candidatus Liberibacter asiaticus* (CLas) and *Candidatus Liberibacter americanus* (CLam), which result in one of the most devastating citrus diseases worldwide, Huanglongbing (HLB) (Duan et al., 2009). HLB causes small and bitter fruits, blotchy mottling, twig dieback, poor root growth, and, ultimately, plant death within 5 years. This disease has resulted in severe economic damage to the Florida citrus industry, with up to $8.9 billion/year in damage since 2006 (Eason et al., 2018). Currently, chemical control is the most effective measure to manage the ACP and HLB because there is a lack of HLB-resistant cultivars. However, the frequent and extensive use of pesticides has also resulted in insecticide resistance in ACP populations. Tiwari et al. (2011) reported that ACP populations from Florida have a high level of resistance to imidacloprid. However, Kanga et al. (2015) showed that ACP populations from Florida in the United States have no resistance to imidacloprid. Pardo et al. (2018) showed that the insecticide resistance of adults and nymphs from ACP populations was significantly different. Tian et al. (2018) found that ACP populations from Guangdong in China have a high level of resistance to imidacloprid. This result indicated that ACP populations from different locations had different resistance levels to the same insecticide, which may result from different resistance mechanisms. Wang et al. (2019) found that the ABC (ATP-binding cassette) transporters of the ACP may affect resistance to imidacloprid. Whether there is a difference in resistance to imidacloprid and thiamethoxam between different ACP populations is unclear. Whether genes associated with acetylcholinesterase and cytochrome P450 mono-oxygenase affected the resistance of ACP from populations in different geographical areas is also unclear.

The ACP harbors the primary endosymbionts *Candidatus Carsonella ruddii* and *Candidatus Profftella armature*, located on the surface and inside of the bacteriome, respectively (Nakabachi et al., 2013). The ACP also harbors secondary symbionts (*Rickettsia*, *Wolbachia*, and *Arsenophonus*), which are located in organs other than the bacteriome (Subandiyah et al., 2000; Saha et al., 2012; Nakabachi et al., 2020). Studies have suggested that *Profftella* and *Wolbachia* affect the acquisition and transmission of *Candidatus Liberibacter* spp. through the toll-signaling pathway, which regulates immunity and resistance (Ramsey et al., 2015; Zhang et al., 2016; Ren et al., 2018). Gandarilla-Pacheco et al. (2013) suggested *B. bassiana* and *Isaria fumosorosea* could be used to biocontrol to ACP in México. However, whether symbiotic bacteria and fungi affect the resistance of the ACP to insecticides is unclear.

In this study, we hypothesized that there are differences in the insecticide resistance level of ACP populations from Hunan and Guangdong to imidacloprid and thiamethoxam and that the resistance differences were affected by the expression of genes associated with cytochrome P450 mono-oxygenase or acetylcholinesterase or the microbial community, either separately or together. To test this hypothesis, we determined (1) the resistance level of A from Hunan and Guangdong to imidacloprid and thiamethoxam, (2) the expression of genes associated with cytochrome P450 mono-oxygenase (*CYP4C67*, *CYP4C68*, *CYP4G70*, *CYP4DA1*, and *CYP4DB1*) and acetylcholine esterase (*AChE -1–like* and *ChE-2–like*, ACH1 and ACH2), and (3) the composition and abundance of the symbiotic bacteria and fungi associated with the ACP.

## Materials and Methods

### Insecticides and Insect Collections

**FIGURE 1:**
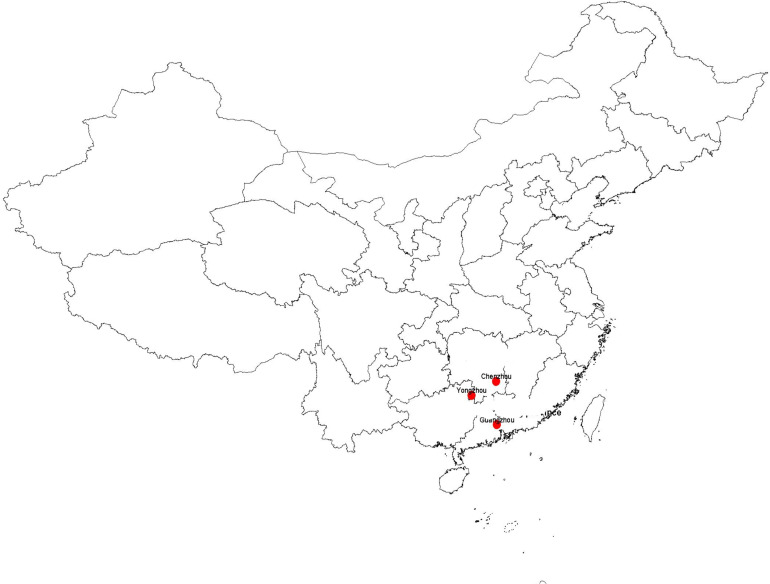
Map of China with red dots indicating locations where the studied samples were collected.

In this study, three geographically distinct populations were collected using aspirators. Two populations were collected from commercial citrus orchards located in Jiangyong County of Yongzhou city (111.33E-25.28N) and Yongxing County of Chenzhou city (113.10E-26.13N) in Hunan Province. The third ACP population came from a commercial *Murraya paniculata* Jack grove located in the Xinfang village of the Liwan district, Guangzhou city (113.23E, 23.16N), Guangdong Province (Figure 1). To enlarge the populations and ensure that all the ACPs were maintained under the same conditions, field ACP populations were cultured for one generation on *M. paniculata* Jack in an air-conditioned glasshouse maintained at 28°C ± 2°C and 70% ± 10% RH under natural light. Technical-grade samples (>98% purity) of imidacloprid and thiamethoxam were purchased from Shanghai Focus Trade Co., Ltd. (Shanghai) and Zhejiang Haizheng Chemical Co., Ltd. (Taizhou), respectively.

### Assessment of ACP Resistance to Insecticides

To compare the difference in resistance between the Hunan and Guangdong ACP populations, the previously described glass vial bioassay technique was used to detect the resistance of ACP to imidacloprid and thiamethoxam (Kanga et al., 2015). Different concentrations of insecticides dissolved in acetone were used to treat the glass scintillation vials (10 mL), and young leaves were soaked for 30 s and dried naturally. Control vials were treated with acetone only. The concentration of imidacloprid was diluted from 80 mg/L to 1.25 mg/L (and 0.625–40 mg/L used in Guangzhou ACP), and the concentration of thiamethoxam was diluted from 80 mg/L to 1.25 mg/L according to 1:2 ratio (twofold). The concentrations were based on preparative experiment. Ten ACPs were placed in each treatment and control vial with fresh young leaves with and without insecticides, respectively, and held at room temperature (28°C ± 1°C and 70% RH; a vial served as a replicate for each concentration tested). Three biological replicates and three technical replicates were performed for each concentration of each insecticide in the study. In total, 1,440 (3 × 3 × 10 × 8 × 2) ACPs were used in the bioassays. Mortality was determined and recorded 24 h after exposure. ACPs unable to walk (>5 mm) after probing with a fine brush were recorded as dead. The resistance difference among the populations from different geographic locations was determined by the resistance ratio (RR) based on the median lethal concentrations (LC_50_) of the highly resistant population divided by the LC_50_ of the low-resistance population. The virulence regression equation, LC_50_, were estimated and the χ^2^ test was performed using DPS software.

### Relative Gene Expression Associated With Resistance to Insecticides

To explore whether changes in gene expression altered ACP resistance to insecticides, we determined the ACP mRNA levels of the genes associated with cytochrome P450 mono-oxygenase (*CYP4C67*, *CYP4C68*, *CYP4DA1*, *CYP4DB1*, and *CYP4G70*) and acetylcholine esterase [*AChE*-1–like (*ACH1*) and *ChE*-2-like (*ACH2*)]. Approximately 100 live ACP individuals/population were used to extract total RNA and to synthesize cDNA at the same time as the bioassay, and resistance was assayed. To further verify the functions of P450 mono-oxygenase in ACP resistance to insecticides, total RNA was extracted, from ACP treated with imidacloprid (LC_50_) after 12, 24, and 48 h. Extraction was performed using Trizol according to the manufacturer’s protocols. The RNA was quantified by a NanoDrop 2000 spectrophotometer (ND 2000) (NanoDrop Technologies Inc., Wilmington, DE, United States). Fifty nanograms of total RNA in a 20-μL volume was used for cDNA synthesis with a transcriptor first-strand cDNA synthesis kit (Trans Gen Biotech, Beijing, China) according to the manufacturer’s protocols. To obtain reliable normalization of the reverse transcriptase–quantitative polymerase chain reaction (RT-qPCR) data, threefold diluted cDNA templates were used to verify each of the primer pairs to find the optimal concentration range for the RT-qPCRs (Hong et al., 2017, 2019). The optimal cDNA concentration was used in each of the RT-qPCR mixtures (10 μL), and three randomly selected optimal cDNA concentrations were used to validate the stability of the housekeeping gene (actin). RT-qPCRs were performed using SYBR Green I (Trans Gen Biotech, Beijing, China) and Bio-Rad software (Bio-Rad, United States). The thermal cycling conditions were as follows: 95°C for 2 min followed by 40 cycles of 95°C for 15 s and Tm temperature for 30 s; this was followed by a dissociation curve analysis, with a ramp-up from 65°C to 95°C and a read every 0.5°C. Ten biological replicates with three technical replicates were performed. The relative quantification of RNA was performed using the Livak method (2^–Δ*CT*^) (Livak and Schmittgen, 2001), and the values obtained for each mRNA were normalized to the ACP Actin. The linear relationship between RR_50_ values and the relative expression of the genes was analyzed.

### The Composition and Abundance of the Microbial Communities

To explore whether changes in the microbial communities affected ACP resistance to insecticides, we determined the composition and abundance of the bacterial and fungal communities from Hunan and Guangdong provinces by 16S and ITS sequencing. Approximately 30 live ACPs/population/time were frozen for 3–5 min (0°C), soaked in 75% (vol:vol) ethanol for 2–3 min, washed three to five times, and used to extract the genomic DNA of the microbe using an insect DNA extraction kit (Mobio, Carlsbad, CA, United States). Sterile water was used as negative controls in the extracted DNA. The quality and concentration of the purified DNA were assessed by an ND 2000 spectrophotometer. The bacterial universal primers 338F (5′-ACTCCTACGGGAGGCAGCA-3′) and 806R (5′-GGACTACHVG GGTWTCTAAT-3′) were used to amplify the V3–V4 region of the bacterial 16S rRNA (Caporaso et al., 2011). The fungal universal primers ITS3-2024 (5′-GCATCGATGA AGAACGCAGC-3′) and ITS4-2409 (5′-TCCTCCGCTTATTGATATGC-3′) were used to amplify the ITS 3/4 region of the fungal ribosomal locus (White et al., 1990). The PCR amplifications were conducted in a 20-μL mixture containing 4 μL of 5 × Fast-Pfu buffer, 2 μL of 2.5 mM dNTPs, 0.8 μL of each primer (5 μM), 0.4 μL of Fast-Pfu polymerase, and 10 ng of template DNA, for which the barcode is an eight-base sequence unique to each sample. The PCR reactions were performed in triplicate (95°C for 2 min followed by 25 cycles at 95°C for 30 s, 55°C for 30 s, and 72°C for 30 s with a final extension at 72°C for 5 min). Amplicons were extracted from 2% agarose gels and purified using an AxyPrep DNA Gel Extraction Kit (Axygen Biosciences, Union City, CA, United States) according to the manufacturer’s instructions and quantified using QuantiFluor^TM^-ST (Promega, United States). The purified amplicons were pooled in equimolar and paired-end sequenced on an Illumina MiSeq PE300 platform according to standard protocols. Raw Fastq files were demultiplexed and quality-filtered using QIIME (version 1.17). The sequencing reads were assigned to each sample according to the unique barcode of each sample, and pairs of reads from the original DNA fragments were merged using FLASH (Martin, 2011). Reads that could not be assembled were discarded, and the number of sequences in each sample was greater than 10,000. Operational taxonomic units (OTUs) were clustered with a 97% similarity cutoff using UPARSE (version 7.1), and chimeric sequences were identified and removed using UCHIME. The taxonomy of each 16S rRNA gene sequence was analyzed by RDP Classifier against the SILVA (SSU115) 16S rRNA database using a confidence threshold that sequences with ≥97% similarity are assigned to the same species, whereas those with ≥95% similarity are assigned to the same genus (Breglia et al., 2010). For each representative sequence from ITS, the Unite Database^1^ was used basing on BLAST algorithm, which was calculated by QIIME software (version 1.9.1)^2^ to annotate taxonomic information using a confidence that sequences with ≥97% similarity are assigned to the same species, whereas those with ≥95% similarity are assigned to the same genus (Breglia et al., 2010). OTU abundance information was normalized using a standard of sequence number corresponding to the sample with the least sequences. The relative abundance was the number of species sequences divided by the total sequences in a sample. The demultiplexed sequence data have been deposited in the National Center for Biotechnology Information^3^ (16s accession PRJNA646487 and ITS accession: PRJNA646485). In this study, 15 microbial samples from three geographical populations (five samples/population) were used to analyze the composition and abundance of the bacterial and fungal communities. We evaluated the differences in the relative abundances of the different geographic populations based on OTUs and species and genus after removing some microbiota that appeared only once and in only one sample. The linear relationship between the RR_50_ value and the mean relative abundance of fungi and the linear relationship between the mean relative abundance of fungi and the mean relative expression of genes were analyzed. To further verify fungi function, the species classification tree based on the top 20 species with the largest relative abundance was constructed. Finally, in the discussion, we inferred the ecological function of the significant fungi based on Fun-Guild and published literatures on fungi detoxification (Luan et al., 2012, 2017; Selvam et al., 2013; Fattahi et al., 2014; Nguyen et al., 2016).

### Statistical Analyses

The percentage of mortality in the treatments was corrected for control mortality by using Abbott’s formula (Abbott, 1925). The virulence regression equation and median lethal concentrations (LC_50_) were estimated, and the χ^2^ test was performed using DPS software. The RR was determined as the LC_50_ of the high-resistance population (Chenzhou or Yongzhou) divided by that of the population with the lowest resistance (Guangzhou). Multivariate analysis and, specifically, a general linear model were used to analyze the relative expression of genes and the relative abundances of the microbial communities; the level of significance was set at *P* < 0.05. The model fit was based on the mean value and the standard deviation (SD) of the corresponding element. The statistical analyses were conducted using SPSS 21.0 (IBM, United States). To further analyze the link between the ACP population resistance (RR_50_) to imidacloprid and the microbiota abundance and gene expression, we established several linear regression equations and determined their correlation coefficients.

## Results

### Assessment of ACP Resistance to Insecticides

**FIGURE 2:**
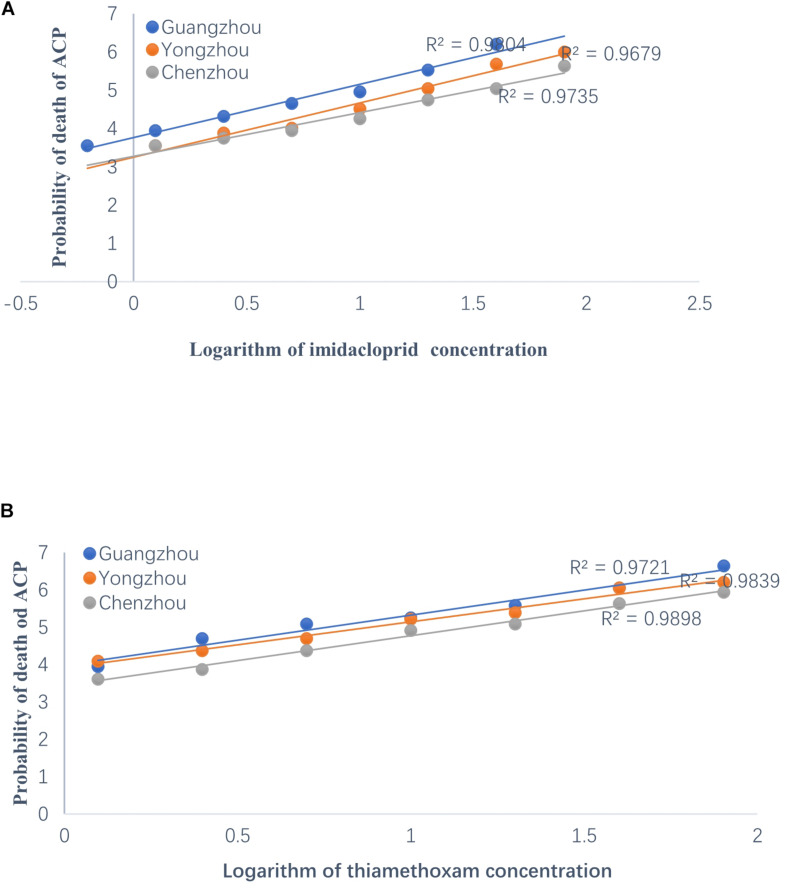
Regression analysis on the logarithm of concentration of insecticides to the probability of death of ACP from Guangzhou, Yongzhou, and Chenzhou. *Y*-axis was the probability of death, which was subjected to probit transformation based on mortality (according to conversion table of biostatistics probability value). The median lethal concentrations (LC_50_) were estimated using DPS software. **(A)** Regression analysis on the logarithm of imidacloprid concentration to the probability of death of ACP. The dilution concentration of imidacloprid was from 40 mg/L to 0.625 mg/L according to 1:2 ratio and the *X*-axis was –0.204, 0.097, 0.398, 0.699, 1, 1.301, 1.602, and 1.903 (logarithmic of imidacloprid concentration). **(B)** Regression analysis on the logarithm of thiamethoxam concentration to the probability of death value of ACP. The dilution concentration of thiamethoxam was from 80 mg/L to 1.25 mg/L according to 1:2 ratio, and the *X*-axis was 0.097, 0.398, 0.699, 1, 1.301, 1.602, and 1.903 (logarithmic of thiamethoxam concentration). Blue dot was for Guangzhou ACP population, orange dot was for Yongzhou ACP population, and gray dot was for Chenzhou ACP population.

The resistance of the ACP to thiamethoxam and imidacloprid appeared to be significantly different among the three geographical populations based on LC_50_ and LC_95_. Regardless of insecticide type (i.e., either thiamethoxam or imidacloprid), at a given concentration, the ACP population from Guangdong Province has the highest probability of mortality and the lowest resistance to thiamethoxam or imidacloprid. The value of the RR for thiamethoxam between the Chenzhou ACP population and Guangzhou ACP population was up to 3.85 for the RR_50_. The value of the RR for thiamethoxam between the Yongzhou ACP population and Guangdong ACP population was 2.18 for the RR_50_. Similarly, the level of resistance of the Hunan ACP populations (Chenzhou and Yongzhou) to imidacloprid was significantly higher than that of the Guangdong ACP population (Table 1 and Figure 2A). And the levels of resistance of Yongzhou and Chenzhou ACP populations to thiamethoxam at LC_50_ were higher than that to imidacloprid (Table 1 and Figure 2).

**TABLE 1:** Toxicity regression equations and χ^2^ test and relative parameter of the logarithm of pesticides concentration to probability of death of different ACP populations exposed to different insecticides.

Insecticide	Population	Toxicity regression equations	LC_50_^a^ (95% CI) (mg/L)	χ^2^	RR_50_^*b*^
Thiamethoxam	Guangzhou	*Y* = 1.466*x* − 1.341	8.214 (6.612–10.388)	7.711	1
	Yongzhou	*Y* = 1.543*x* − 1.932	17.866 (14.481–22.445)	8.769	2.18
	Chenzhou	*Y* = 1.331*x* − 1.996	31.6 (24.388–43.422)	9.046	3.85
Imidacloprid	Guangzhou	*Y* = 1.253*x* − 0.957	5.802 (4.541–7.459)	9.626	1
	Yongzhou	*Y* = 1.222*x* − 1.452	15.408 (11.857–19.766)	5.623	2.66
	Chenzhou	*Y* = 1.339*x* − 1.976	29.9 (23.740–38.349)	8.141	5.16

*^*a*^LC_50_ values followed by different letters within each group were significantly different from one another, based on non-overlapping of 95% confidence limit. ^a^LC_50_ values were measured for each insecticide and population. CL, confidence limit. ^*b*^RR (resistance ratios) = LC_50_ of (Yongzhou or Chenzhou) ACP/LC_50_ of Guangzhou ACP.*

### Relative Gene Expression Associated With Resistance to Insecticides

**FIGURE 3:**
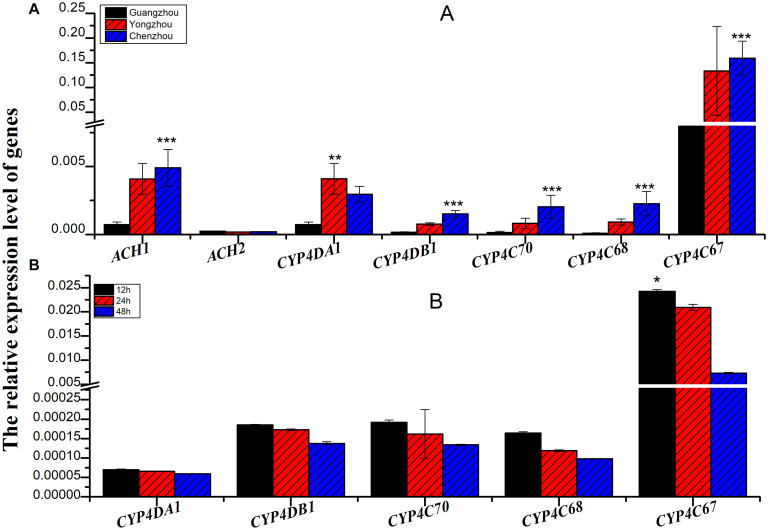
The relative expression of genes associated with cytochrome P450 mono-oxygenase (*CYP4C67*, *CYP4C68*, *CYP4DA1*, *CYP4DB1*, and *CYP4G70*) and acetylcholine esterase [*AChE*-1-like (*ACH1*) and *ChE*-2-like (*ACH2*)]. **(A)** The relative expression of genes associated with cytochrome P450 mono-oxygenase and acetylcholine esterase from different ACP populations. **(B)** The relative expression of genes associated with cytochrome P450 mono-oxygenase from Guangzhou ACP at different times after LC_50_ imidacloprid treatment. Black bar was from Guangzhou ACP population, red bar was from Yongzhou ACP population, and blue bar was from Chenzhou ACP population *P ≤ 0.05, **P ≤ 0.01, ***P ≤ 0.001.

The relative expression of all the genes except *ACH2* was significantly different among the three populations, and the relative expression of all the genes was the lowest in the Guangzhou population. In fact, among the three populations, extremely significant differences were found for *CYP4C67* [*F*_(__2_,_27__)_ = 21.056, *P* < 0.001], *CYP4C68* [*F*_(__2_,_27__)_ = 14.979, *P* < 0.001], *CYP4C70* [*F*_(__2_,_27__)_ = 28.084, *P* < 0.001], and *CYP4DB1* [*F*_(__2_,_27__)_ = 19.673, *P* < 0.001]. *CYP4DA1* [*F*_(__2_,_27__)_ = 6.991, *P* = 0.004] was moderately significantly different (Figure 3A and Supplementary Table S1). To further verify the effect of P450 on ACP resistance to insecticides, we determined the relative expression of P450 in the Guangzhou population during treatment with semilethal doses

**S1:** The relative expression level of ACP genes.

Pop gene	Guangzhou	SD	Chenzhou	SD	Yongzhou	SD
*ACTIN*	1	0.07060	1	0.07009	1	0.090600
*ACH1*	0.000726	0.000186	0.004910	0.001350	0.004093	0.001129
*ACH2*	0.000238	0.000014	0.000197	0.000027	0.000187	0.000019
*CYP4DA1*	0.000726	0.000187	0.002962	0.000585	0.004094	0.001132
*CYP4DB1*	0.000162	0.000040	0.001518	0.000249	0.000756	0.000108
*CYP4C70*	0.000152	0.000082	0.002035	0.000853	0.000825	0.000374
*CYP4C68*	0.000095	0.000023	0.002261	0.000897	0.000919	0.000220
*CYP4C67*	0.013508	0.013534	0.158878	0.034592	0.133441	0.089725

(LC_50_) of imidacloprid for 12–48 h. The relative expression of all the genes was up-regulated, and the highest relative expression occurred after 12 h. However, the differences among four of the genes (excluding *CYP4C67*) were not significant (Figures 3B, Supplementary Table S2). Moreover, positive linear relationships between the RR_50_ value and relative expression of *CYP4DB1* and *CYP4C70* were observed, and positive linear relationships between the relative abundance of *Aspergillus niger* and *Aureobasidium pullulans* and relative expression of *CYP4DB1* and *CYP4C70* were observed (Figures 4C–H).

**S2:** The relative expression level of ACP genes in different time.

Time gene	12 h	SD	24 h	SD	48 h	SD
*CYP4DA1*	0.0000699	0.0000017	0.0000658	0.0000005	0.0000591	0.0000007
*CYP4DB1*	0.0001853	0.0000013	0.0001730	0.0000019	0.0001374	0.0000042
*CYP4C70*	0.0001921	0.0000056	0.0001613	0.0000635	0.0001338	0.0000014
*CYP4C68*	0.0001642	0.0000035	0.0001188	0.0000023	0.0000980	0.0000009
*CYP4C67*	0.0242528	0.0003412	0.0209174	0.0006373	0.0072965	0.0001502

**FIGURE 4:**
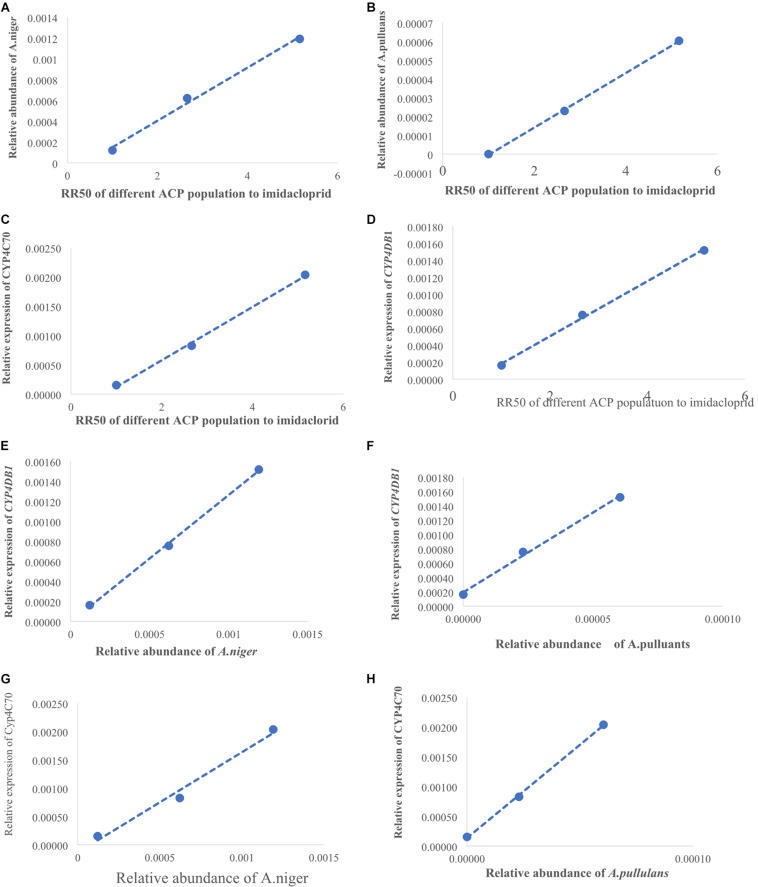
The positive linear correlations picture. **(A)** Linear relationship between RR_50_ of imidacloprid and the relative abundance of *A. niger*. **(B)** Linear relationship between RR_50_ of imidacloprid and the relative abundance of *A. pullulans*. **(D)** Linear relationship between RR_50_ of imidacloprid and relative expression of *CYP4DB1*. **(C)** Linear relationship between RR_50_ of imidacloprid and relative expression of *CYP4C70*. **(E)** Linear relationship between *A. niger* and *CYP4DB1*. **(G)** Linear relationship between *A. niger* and *CYP4C70*. **(F)** Linear relationship between *A. pullulans* and *CYP4DB1*. **(H)** Linear relationship between *A. pullulans* and *CYP4C70*. *X*-axis of panel **(A–D)** shows the RR_50_ value; the first dot was for Guangzhou ACP resistance (=1), the second dot was for Yongzhou ACP RR_50_, and the third dot was for Chenzhou ACP RR_50_. *X*-axis of panel **(E–H)** was relative abundance of fungi; the first dot was for Guangzhou ACP, the second dot was for Yongzhou ACP, and the third dot was for Chenzhou ACP. *Y*-axis of panels **(A,B)** shows the relative abundance of fungi, and *Y*-axis of panels **(D,H)** shows the relative expression of genes.

### The Composition and Abundance of the Microbial Communities

A total of 528 OTUs were generated, and 315 genus and 152 species were annotated in all samples by 16S sequencing. The known primary symbiont (*Candidatus Profftella*) and secondary symbionts (*Wolbachia*) of ACP were assigned by our analytical procedure for the 16S amplicon sequence. And the relative mean abundance of primary symbiont was more than 0.8 (≥80%), the relative mean abundance of secondary symbiont was more than 0.1(≥10%). One hundred sixteen OTUs were unique to Guangzhou ACP population, 134 OTUs were unique to Yongzhou ACP population, and 39 OTUs were unique to Chenzhou ACP population (Venn diagram Supplementary Figure S1).


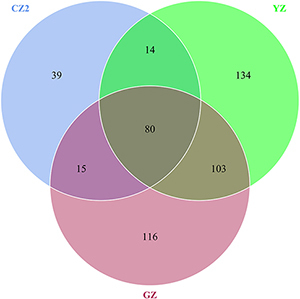


A total of 1,008 OTUs were generated, and 239 genus and 265 species were annotated in all samples by ITS sequencing. One hundred thirteen OTUs were unique to Guangzhou ACP population, 147 OTUs were unique to Yongzhou ACP population, and 117 OTUs were unique to Chenzhou ACP population (Venn diagram Supplementary Figure S2).


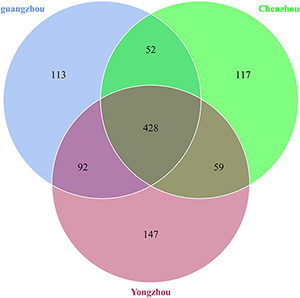


**FIGURE 5:**
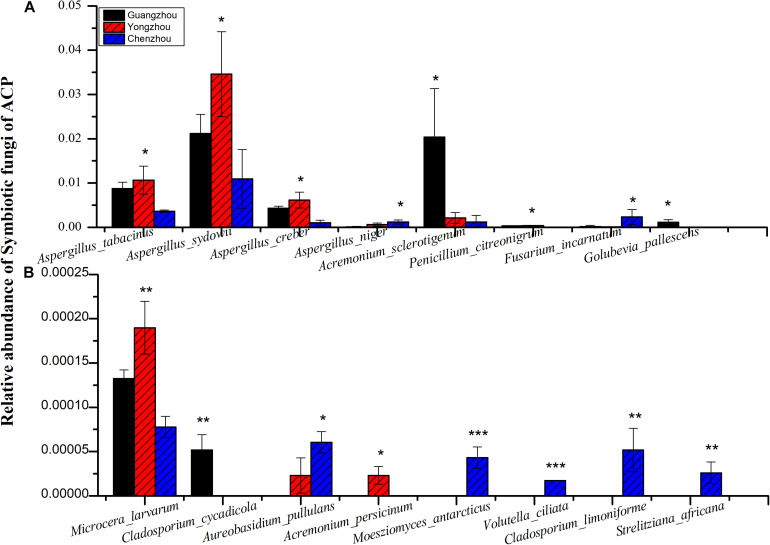
Comparing the relative abundance of the fungi from different ACP populations. Black bar was from Guangzhou ACP population, red bar was from Yongzhou ACP population, blue bar was from Chenzhou ACP population. **(A)** Relative high abundance fungi. **(B)** Relative low abundance fungi. *Y*-axis shows the relative abundance (0–1) of symbiotic fungi of ACP; that is, the OTUs of every fungus were divided by total OTUs. *X*-axis shows the name of the fungi. *P ≤ 0.05, **P ≤ 0.01, ***P ≤ 0.001.

**FIGURE 6:**
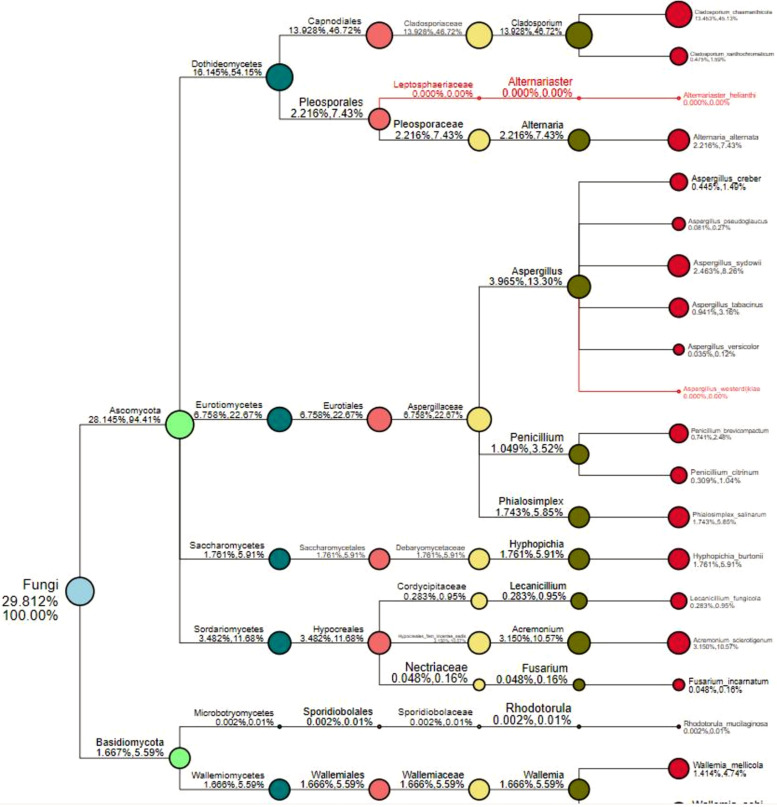
Species classification tree. The circles of different colors indicate different classification levels, corresponding to the legend on the left; the size of the circle represents the relative abundance of the category; the two numbers below the category name both indicate the relative abundance percentage; the former indicates that the category occupies the sample. The percentage of all classified species in the sample; the latter indicates the percentage of the classification in the classified species selected in the sample. The classification in red font indicates that the classification annotation does not exist in the sample, but it exists in other analyzed samples.

The numbers of unique OTUs in bacteria and fungi from Yongzhou ACP populations was the biggest, and that from Chenzhou ACP populations was the smallest in bacteria, and that from Guangzhou ACP population was the smallest in fungi (Supplementary Figures S1, S2). At the same time, 59.5% OTUs were unnamed species in fungi, and 90.2% OTUs were unnamed species in bacteria. Although the numbers of OTUs from 16S sequencing among three ACP populations were different, but the difference level of composition and abundance of bacteria at species or genus level was not significant. For example, the high difference of four bacteria from three ACP populations, *Staphylococcus sciuri* [*F*_(__2_,_12__)_ = 4.638, *P* = 0.073], *Brevibacterium epidermidis* [*F*_(__2_,_12__)_ = 4.937, *P* = 0.066], *Ileibacterium valens* [*F*_(__2_,_12__)_ = 4.597, *P* = 0.074], and *Bacteroides acidifaciens* [*F*_(__2_,_12__)_ = 5.031, *P* = 0.063], were marginally significantly different (0.05 < *P* < 0.1). However, the mean abundances of sixteen fungi were significantly different among the three ACP populations. Among them, four fungi were unique to the Chenzhou ACP population, and only one fungus was unique to the Guangzhou and Yongzhou ACP populations (Table 2 and Figure 5). Moreover, positive linear correlations were also observed between the mean abundances of two fungi and ACP resistance to pesticides (Figures 4A,B), and negative correlations were observed between only the mean abundances of two fungi and ACP resistance to pesticides, and positive linear correlations were also observed between the mean abundances of two fungi and the mean expression of two genes (Figures 4E–H). The species classification tree (Figure 6) showed *Aspergillus*, *Acremonium*, *Penicillium*, and *Cladosporium* were of high abundance and may be associated with ACP resistance. Recently, we assessed *A. niger* and *A. pullulans* resistance to pesticides by plate confrontation *in vitro*. The result showed *A. niger* and *A. pullulans* could grow in PDA with up to 160 mg/L imidacloprid and up to 96 mg/L thiamethoxam, and the concentration of pesticides negatively affected the diameter of fungi disk in 48 h, and the diameters of fungi disk grown on treatment and control PDA were not different after 72 h (Supplementary Figure S3).


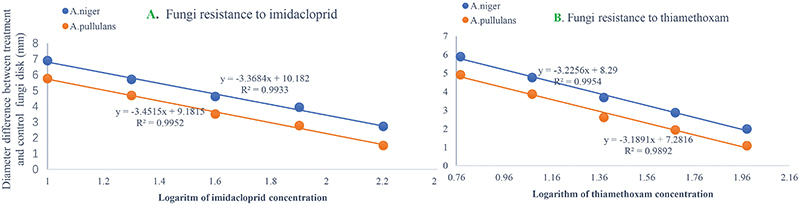


**TABLE 2:** ANOVA results (*P-*values) for the difference level of three ACP populations.

Pop fungi	Guangzhou	*SD*	Yongzhou	*SD*	Chenzhou	*SD*	*P*
*Aspergillus_ tabacinus*	0.00874	0.00027	0.01063	0.00316	0.00364	0.00147	0.045
*Aspergillus_ sydowii*	0.02120	0.00660	0.03458	0.0096	0.01093	0.00429	0.039
*Aspergillus_ creber*	0.00433	0.00058	0.00616	0.00177	0.00100	0.00043	0.014
***Aspergillus_ niger***	**0.00012**	**0.00044**	**0.00062**	**0.00035**	**0.00119**	**7.91E-05**	**0.031**
***Aureobasidium_ puUulans***	**0**	**1.22E-05**	**2.30E-05**	**1.99E-05**	**6.04E-05**	**0**	**0.013**
***Acremonium_sc lerotigenum***	**0.02038**	**0.00148**	**0.00212**	**0.00120**	**0.00122**	**0.01096**	**0.039**
***Golubevia _ pallescens***	**0.00116**	**1.22E-05**	**2.88E-05**	**4.98E-05**	**0.00058**	**8.63E-06**	**0.022**
Penicillium_ citreonigrum	0.00032	4.88E-05	0.00037	9.8152E-05	8.16E-05	3.45E-05	0.015
*Fusarium_ incarnatum*	0.00018	0.00172	7.48E-05	3.59E-05	0.00232	0.00026	0.049
*Microcera_ larvarum*	0.00013234	1.22E-05	0.00019	2.99E-05	7.77E-05	9.97E-06	0.005
*Cladosporium_c ycadicola*	5.18E-05	0	0	0	1.73E-05	0	0.004
*Acremonium_pe rsicinum*	0	0	2.30E-05	9.97E-06	0	0	0.011
***Moesziomyces_a ntarcticus***	**0**	**1.22E-05**	**0**	**0**	**4.32E-05**	**0**	**0.0005**
***Volutella_ ciliata***	**0**	**0**	**0**	**0**	**1.73E-05**	**0**	**0**
***Cladosporium_l imoniforme***	**0**	**2.44E-05**	**0**	**0**	**5.18E-05**	**0**	**0.006**
***Strelitziana_ africana***	**0**	**1.22E-05**	**0**	**0**	**2.59E-05**	**0**	**0.006**

*Bold font indicates positive or negative correlation with resistance level; the blue was unique to Chenzhou ACP population.*

## Discussion

As a notorious Rutaceae plant pest, ACP not only affects the growth of young foliage by direct feeding but also transmits *C. Liberibacter asiaticus* (Las) and *C. Liberibacter americanus* (Lam), resulting in one of the most devastating citrus diseases worldwide, HLB (Duan et al., 2009; Gottwald, 2010; Grafton-Cardwell et al., 2013; Shimwela et al., 2016). Organophosphates, carbamates, pyrethroids, phenylpyrazoles, and neonicotinoids have been used extensively to control all kinds of insect pests, resulting in different resistance levels to major pesticides (Wang et al., 2009; Wen et al., 2009). In this study, we found that the ACP population in China had resistance to imidacloprid and thiamethoxam and that the Hunan ACP populations had higher resistance levels than the Guangzhou ACP population. The results were consistent with previous studies showing that ACP populations from different geographic locations had different levels of resistance to imidacloprid (Tiwari et al., 2011; Kanga et al., 2015; Tian et al., 2018).

Many studies have also shown that increased activities of general esterase and P450 mono-oxygenase are common insecticide resistance mechanisms against organophosphate, pyrethroid, and neonicotinoid insecticides in some insect pests (Cao et al., 2008; Zhang et al., 2014; Garrood et al., 2016). Studies have shown that enhanced expression of genes associated with cytochrome P450 mono-oxygenase contributes to neonicotinoid resistance in BPHs (Puinean et al., 2010; Bass et al., 2011; Tian et al., 2018). In this study, the positive linear relationship between the relative expression of two genes (*CYP4C70* and *CYP4DB1*) associated with P450 mono-oxygenase and the ACP RR_50_ to imidacloprid and the positive linear relationship between the relative expression of *CYP4C70* and *CYP4DB1* and the relative abundance of *A. niger* and *A. pullulans* were observed, suggesting *A. niger* and *A. pullulans* and P450 together affected ACP resistance to imidacloprid and thiamethoxam. Moreover, the relative expression of all the genes from the Guangzhou population, which is associated with cytochrome P450, increased after insecticide treatment; the expression level was highest 12 h after treatment and then declined. The results were similar with literature that cytochrome P450 mono-oxygenase was mainly involved in ACP resistance to imidacloprid and thiamethoxam (Guitard et al., 2014; Tian et al., 2018). The literature shows that *CYP6ER1* affects the resistance of *N. lugens* to imidacloprid, thiamethoxam, buprofezin, and ethiprole (Wu et al., 2018), whereas in this study, *CYP4C70* and *CYP4DB1* affected ACP resistance to imidacloprid and thiamethoxam; these findings may be due to the same enzyme being regulated by different microbiology in different species.

The literature also shows that bacteria such as Stenotrophomonas maltophilia, Bacillus licheniformis, Bacillus megaterium, Rahnella aquatilis, and fungi such as Umbelopsis isabellina, V. ciliata, and Botrytis cinerea can degrade atrazine, which is the most commonly detected pesticide in food and drinking water (Marecik et al., 2008; Singh et al., 2018). In this study, the relative abundance of all the bacteria among the three ACP populations was not significant. This result suggested that the symbiotic bacteria of the ACP were not similar to those of Wolbachia in the whitefly and the mosquito Culex pipiens L. (Diptera: Culicidae), which may be involved in host’s resistance to imidacloprid and thiamethoxam (Ghanim and Kontsedalov, 2009). Furthermore, there were significant differences in the resistance mechanisms of different species to the same pesticides (Kanga et al., 2015; Tian et al., 2018; Wang et al., 2019). Moreover, that there was not significantly different bacterial community among ACP populations at species level may be due to 90.2% OTUs being unnamed species in bacteria.

FunGuild is a fungal environmental function database. Based on the support of existing literature, the ecological function of fungi is classified, and the FunGuild database is constructed. Based on species information obtained from amplicon analysis, the ecological functions of existing species in the literature can be queried in the environment. Because FunGuild was mainly used in analysis on environmental microbiology and limited to existing literature, the fungi from ACP were associated plant pathogen and animal pathogen (Gandarilla-Pacheco). Although FunGuild analysis did not find significantly different fungi that were associated with ACP resistance to pesticides, yet the species classification tree (Figure 6) showed *Aspergillus*, *Acremonium*, *Penicillium*, and *Cladosporium* were dominant strains.

*A. pullulans* and *A. niger* can exist in different agroecological niches, and they produce a neutral polysaccharide, antimycotic aureobasidin, antibacterial compounds, melanin, liamocins, siderophore, and extracellular enzymes such as P450 (Franken et al., 2014; Molnárová et al., 2014; Prasongsuk et al., 2018). Polysaccharides, antimycotic aureobasidins, antibacterial compounds, and melanin play important roles in insect immune and defense systems (Luan et al., 2012, 2017; Selvam et al., 2013). P450 was involved in insecticide resistance of pest (Li et al., 2010; Puinean et al., 2010; Safi et al., 2017; Tian et al., 2018; Wang et al., 2019). Although the role of symbiotic fungi in the degradation of insecticides has not been studied extensively, roles of *A. niger* in the immunity and growth of insect and animal have been reported (Medeiros et al., 2009; Fattahi et al., 2014). In this study, four *Aspergillus* species, *A. tabacinus*, *A. sydowii*, *A. creber*, and *A. niger*, were significantly different among the three populations. Positive linear correlations were observed between the relative mean abundances of *A. niger* and *A. pullulans* and ACP resistance to imidacloprid, and positive linear correlations were also observed between the relative mean abundances of *A. niger* and *A. pullulans* and the relative mean expression of *CYP4DB1* and *CYP4C70*. Recently, we assessed *A. niger* and *A. pullulans* resistance to pesticides by plate confrontation *in vitro*. The result showed *A. niger* and *A. pullulans* could grow in PDA with 160 mg/L imidacloprid and 96 mg/L thiamethoxam, and the concentration of pesticides negatively affected the diameter of fungi disk in 48 h, and the diameters of fungi disk grown on treatment and control PDA were not different after 72 h (Supplementary Figure S3). These findings suggested that *A. niger* and *A. pullulans* may be positively affect ACP resistance, and *G. pallescens* and *A. sclerotigenum* may be negatively affect ACP resistance. Whether ACP symbiotic fungi affect insect host resistance to pesticides by producing compounds associated with ACP immunity, such as polysaccharide, antimycotic aureobasidin, antibacterial compounds, melanin, liamocins, siderophore, or producing P450 to degrade pesticides, remains to be elucidated, which will be our work in the future. Moreover, four fungi, *Moesziomyces antarcticus*, *V. ciliata*, *Cladosporium limoniforme*, and *Strelitziana africana*, were unique to the Chenzhou population, which had the highest resistance level among the ACP populations. Whether they were associated with ACP resistance needs further study.

## Data Availability Statement

The demultiplexed sequence data have been deposited in National Center for Biotechnology Information (https://www.ncbi.nlm.nih.gov/sra, 16s accession PRJNA646487 and ITS accession PRJNA646485).

## Author Contributions

TY and YH designed the experiment and wrote the manuscript. LL, LH, and JY performed the experiment. LL and LD analyzed data and drew dragram. All authors contributed to the article and approved the submitted version.

## Conflict of Interest

The authors declare that the research was conducted in the absence of any commercial or financial relationships that could be construed as a potential conflict of interest.

## Footnotes

1 https://unite.ut.ee

2 http://qiime.org/scripts/assigntaxonomy.html

3 https://www.ncbi.nlm.nih.gov/sra
