# Supplementary material for: Symbiotic Fungus Affected the Asian Citrus Psyllid (ACP) Resistance to Imidacloprid and Thiamethoxam
Source: Front Microbiol. 2020 Dec 16;11:522164. doi: 10.3389/fmicb.2020.522164 (PMC7772971; doi:10.3389/fmicb.2020.522164)
Supplement: Supplementary file 1 [file Data_Sheet_1.pdf]

S1 The relative expression level of ACP genes

gene \ pop	**Guangzhou**	**SD**	**Chenzhou**	**SD**	**Yongzhou**	**SD**
ACTIN	1	0.07060	1	0.07009	1	0.090600
ACH1	0.000726	0.000186	0.004910	0.001350	0.004093	0.001129
ACH2	0.000238	0.000014	0.000197	0.000027	0.000187	0.000019
CYP4DA1	0.000726	0.000187	0.002962	0.000585	0.004094	0.001132
CYP4DB1	0.000162	0.000040	0.001518	0.000249	0.000756	0.000108
CYP4C70	0.000152	0.000082	0.002035	0.000853	0.000825	0.000374
CYP4C68	0.000095	0.000023	0.002261	0.000897	0.000919	0.000220
CYP4C67	0.013508	0.013534	0.158878	0.034592	0.133441	0.089725
