# Supplementary material for: Symbiotic Fungus Affected the Asian Citrus Psyllid (ACP) Resistance to Imidacloprid and Thiamethoxam
Source: Front Microbiol. 2020 Dec 16;11:522164. doi: 10.3389/fmicb.2020.522164 (PMC7772971; doi:10.3389/fmicb.2020.522164)
Supplement: Supplementary file 2 [file Data_Sheet_2.pdf]

S2 The relative expression level of ACP genes in different time

time / gene	**12h**	**SD**	**24h**	**SD**	**48h**	**SD**
CYP4DA1	0.0000699	0.0000017	0.0000658	0.0000005	0.0000591	0.0000007
CYP4DB1	0.0001853	0.0000013	0.0001730	0.0000019	0.0001374	0.0000042
CYP4C70	0.0001921	0.0000056	0.0001613	0.0000635	0.0001338	0.0000014
CYP4C68	0.0001642	0.0000035	0.0001188	0.0000023	0.0000980	0.0000009
CYP4C67	0.0242528	0.0003412	0.0209174	0.0006373	0.0072965	0.0001502
