# Supplementary figures and images for: Symbiotic Fungus Affected the Asian Citrus Psyllid (ACP) Resistance to Imidacloprid and Thiamethoxam
Source: Front Microbiol. 2020 Dec 16;11:522164. doi: 10.3389/fmicb.2020.522164 (PMC7772971; doi:10.3389/fmicb.2020.522164)

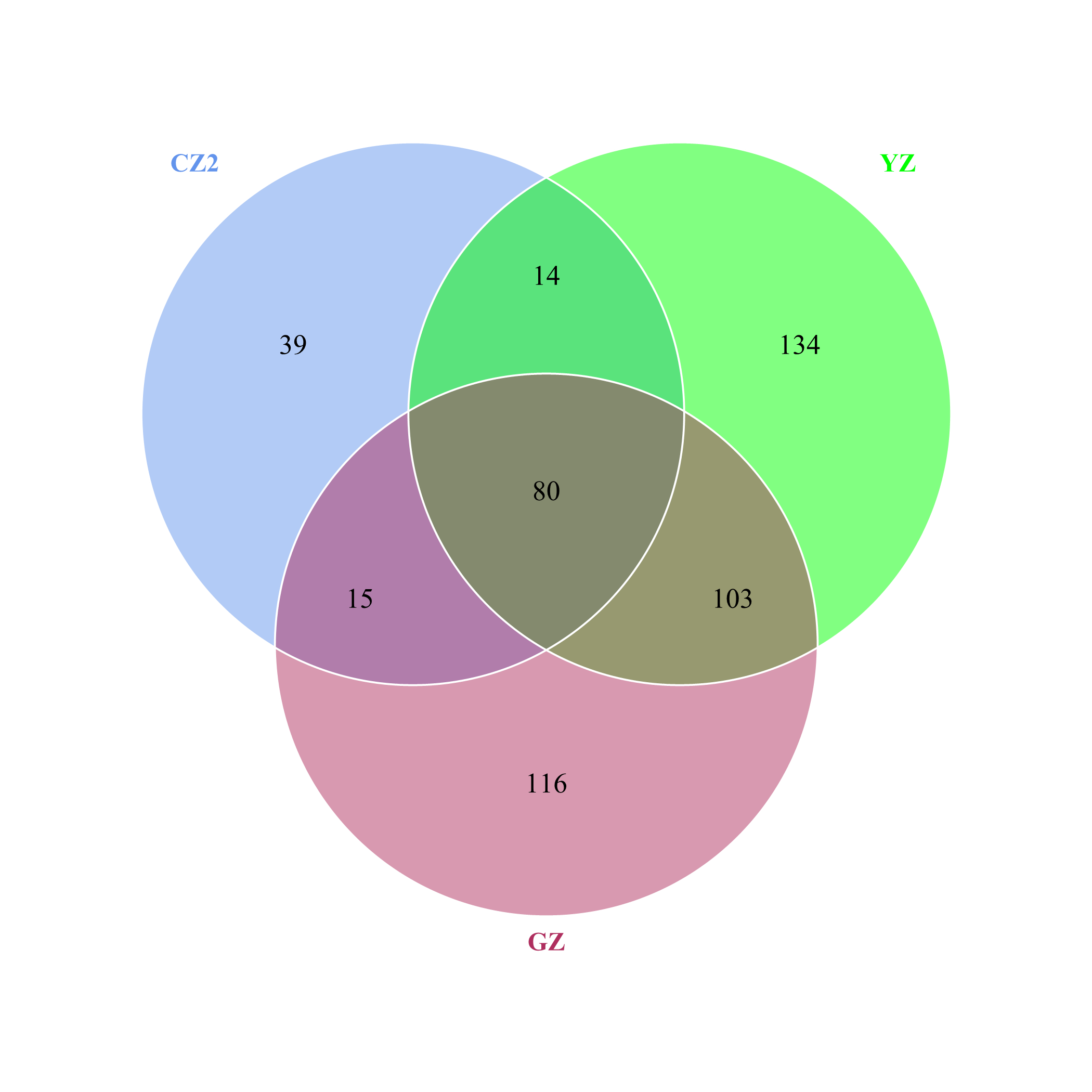

Supplement: Supplementary file 3 [file Image_1.TIF]

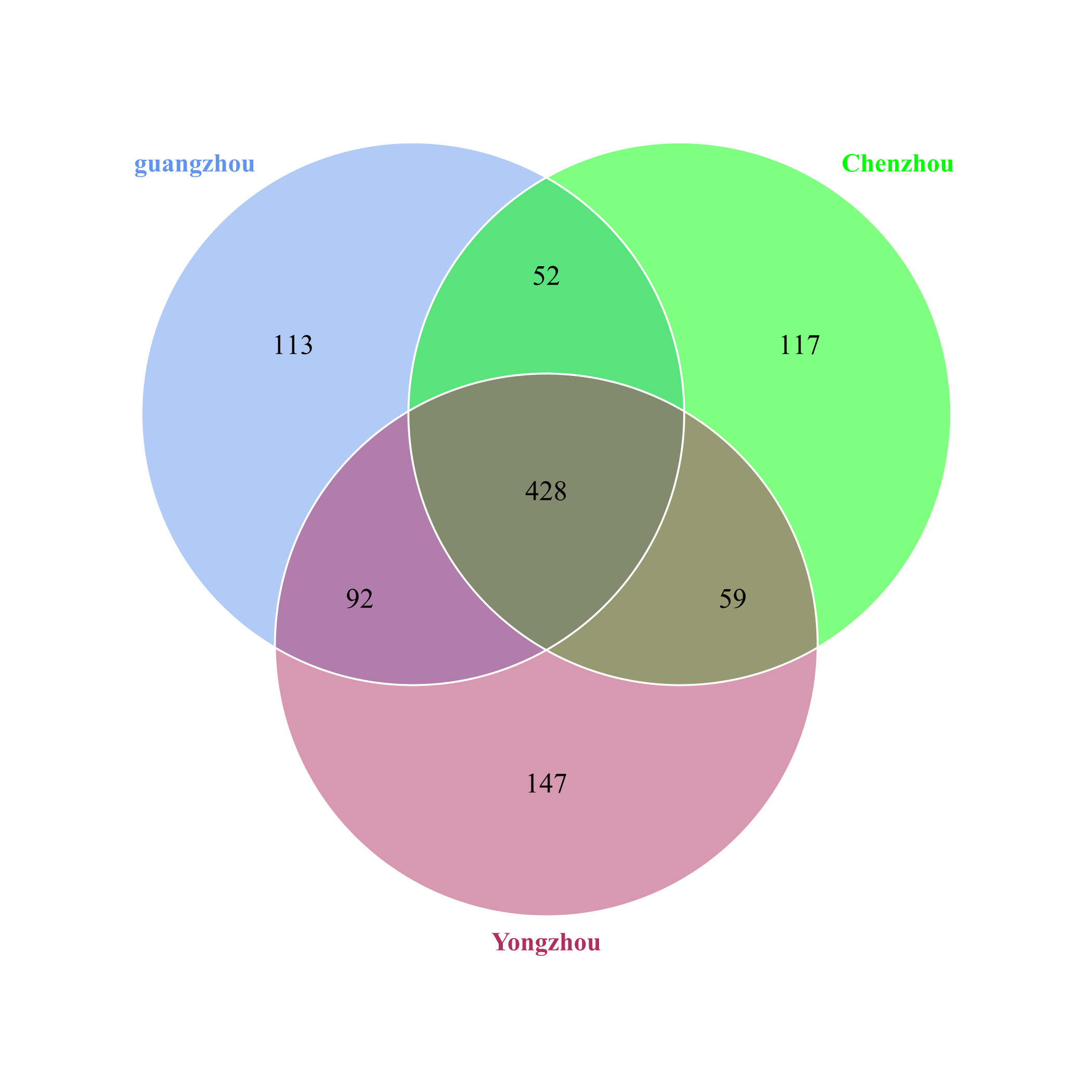

Supplement: Supplementary file 4 [file Image_2.TIF]

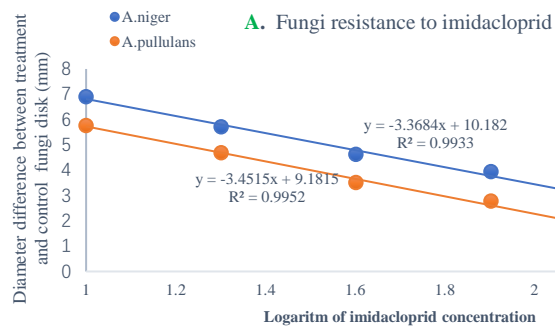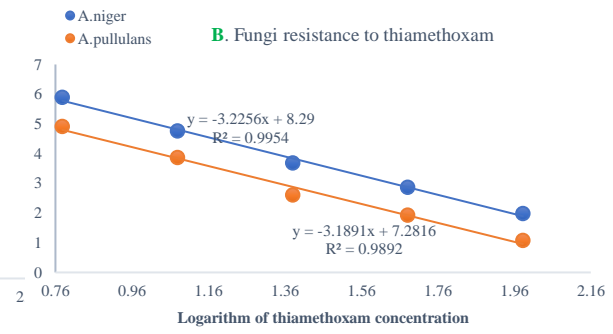

Supplement: Supplementary file 5 [file Image_3.pdf]
